# Modelling of *p*-tyramine transport across human intestinal epithelial cells predicts the presence of additional transporters

**DOI:** 10.3389/fphys.2022.1009320

**Published:** 2022-11-10

**Authors:** Shreyasi Sarkar, Ivan Saika-Voivod, Mark D. Berry

**Affiliations:** ^1^ Department of Biochemistry, Memorial University of Newfoundland, St. John’s, NL, Canada; ^2^ Department of Physics and Physical Oceanography, Memorial University of Newfoundland, St. John’s, NL, Canada

**Keywords:** *p*-tyramine, trace amines, transporters, intestinal epithelium, simulations, kinetics, Caco-2 cells

## Abstract

*p*-Tyramine (TYR) is an endogenous trace amine, which can also be synthesized by intestinal microbiota, and is present in commonly consumed diets. TYR is an agonist for the intracellular trace amine-associated receptor 1, which has been implicated in psychiatric, metabolic, and immune-related disorders. We have previously demonstrated TYR readily diffuses across lipid bilayers, while transport across Caco-2 cell membranes involves Organic Cation Transporter 2 (OCT2) and a Na^+^-dependent active transporter. Here we developed mathematical models to determine whether known kinetics for these processes are sufficient to explain observed transcellular TYR passage. Ordinary differential equations were developed for known TYR transport processes to predict concentration-time relationships. Michaelis-Menten kinetics were assumed for all transporter-mediated processes and a one phase exponential function used for simple diffusion. Modelled concentration-time plots were compared to published experimental results. Additional transporter functions were sequentially added to models to improve consistency, and a least squares error minimization approach utilized to determine added transporter kinetics. Finally, possible TYR compartmentalization was also modelled. Following apical loading, transport across the apical, but not the basolateral, membrane was modelled without additional transporters, suggesting a basolateral transporter was missing. Consistent with this, models of basolateral compartment loading did not match experimental observations, indicating missing basolateral transporters were bidirectional. Addition of a transporter with the kinetic characteristics of OCT2 did not improve models. Varying the kinetic parameters of the added transporter improved models of basolateral, but worsened apical, loading models, suggesting the need for either a directional preference in transporters, or intracellular TYR compartmentalization. Experimental parameters were recapitulated by introducing asymmetry into the apical OCT2 (K_t_OCT2_apicaltocell_ = 110.4 nM, K_t_OCT2_celltoapical_ = 1,227.9 nM), and a symmetric basolateral facilitated diffusion transporter (V_max_ = 6.0 nM/s, K_t_ = 628.3 nM). The apparent directionality of OCT2 may reflect altered TYR ionization due to known pH differences between compartments. Models for asymmetry and compartmentalization were compared by root mean square deviation from experimental data, and it was found that TYR compartmentalization could only partially replace the need for asymmetry of OCT2. In conclusion, modelling indicates that known TYR transport processes are insufficient to explain experimental concentration-time profiles and that asymmetry of the apical membrane OCT2 combined with additional, low affinity, basolateral membrane facilitated diffusion transporters are required.

## 1 Introduction

*p*-Tyramine (TYR) is a member of the trace amine (TA) family that has very low endogenous tissue concentrations (<10 ng/g; 100 nM), at least 100-fold lower than the classical monoamine neurotransmitters (Berry, 2004). Endogenous production of TYR takes place by the action of aromatic L-amino acid decarboxylase (EC 4.1.1.28) mediated decarboxylation (Berry et al., 2017) of L-tyrosine. Other sources of TYR include the intestinal microbiota (Wolken et al., 2006; Landete et al., 2007; Bonnin-Jusserand et al., 2012; Mazzoli and Pessione, 2016; Yang et al., 2016; Fujisaka et al., 2018) and common dietary items including red wine (Nalazek-Rudnicka and Wasik, 2017), chocolate (Martin and Vij, 2016), aged cheeses (Liu et al., 2018), fermented meats (Zhang et al., 2019), and seafood (An et al., 2015; Biji et al., 2016). TYR is an agonist at trace amine-associated receptor 1 (TAAR1) (Bonner et al., 2019), a G protein-coupled receptor that was discovered in 2001 (Borowsky et al., 2001; Bunzow et al., 2001) and has since been established as a therapeutic target for psychiatric (Berry et al., 2017; Nazimek J et al., 2017; Koblan et al., 2020; Nair et al., 2022), metabolic (Revel et al., 2012; Adriaenssens et al., 2015; Ferragud et al., 2016; Raab et al., 2016; Michael et al., 2019; Cripps et al., 2020) and immune-related disorders (Christian and Berry, 2018; Barnes et al., 2021). Accordingly, TAAR1 has been shown to be present in various cells in the brain (Borowsky et al., 2001; Lindemann et al., 2005; Revel et al., 2012; Cisneros and Ghorpade, 2014), spinal cord (Borowsky et al., 2001; Lindemann et al., 2008; Gozal et al., 2014), pancreatic β cells, stomach D cells (Chiellini et al., 2012; Revel et al., 2012; Adriaenssens et al., 2015; Raab et al., 2016), intestinal enterochromaffin mucosal cells (Kidd et al., 2008; Ito et al., 2009; Revel et al., 2012; Raab et al., 2016), and human leukocytes (D’Andrea et al., 2003; Nelson et al., 2007; Babusyte et al., 2013; Barnes et al., 2021). In all these cell types, TAAR1 exhibits a primarily intracellular localization (Lindemann et al., 2005; Barak et al., 2008; Pei et al., 2016; Raab et al., 2016; Barnes et al., 2021). It is important, therefore, to understand the transport properties of TYR across cell membranes to access TAAR1. Further, such transport processes in intestinal epithelial cells will be relevant to microbiota and food-derived TYR interaction with cells of the immune system (Christian and Berry, 2018).

**FIGURE 1:**
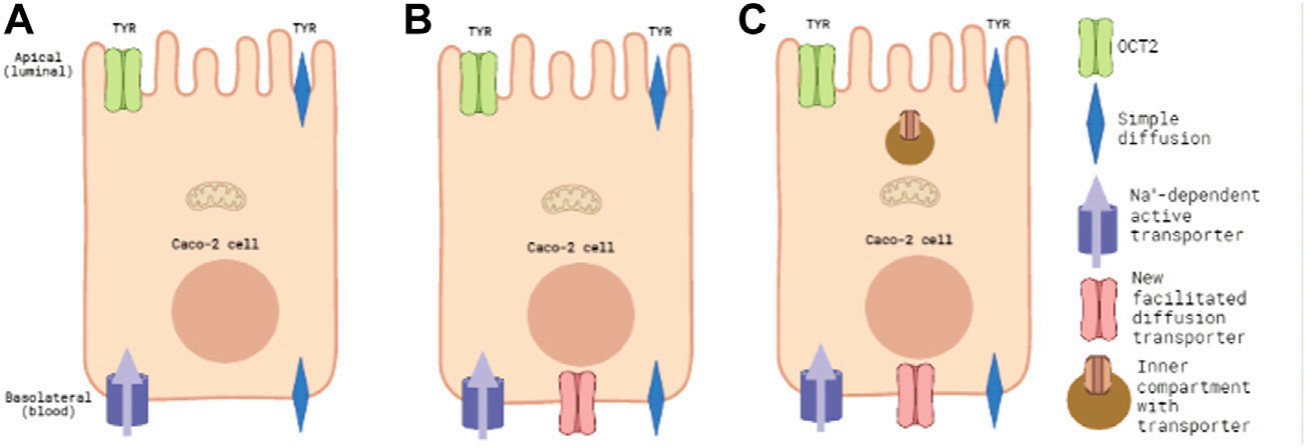
Known and predicted TYR transporters used for modelling transcellular passage in Caco-2 cells. Panel 1**(A)** represents the baseline model of known TYR transport processes in the Caco-2 intestinal cell line, where TYR can cross Caco-2 plasma membranes by simple diffusion, be transported across the apical membrane by the bi-directional facilitated diffusion transporter OCT2, or actively accumulated within the cell from the basolateral compartment by an as yet unidentified Na^+^-dependent active transporter. Panel 1**(B)** represents the model where an additional facilitated diffusion transporter in the basolateral membrane has been added and optimal kinetic parameters that maximized consistency with experimental concentration-time relationships determined. Panel 1**(C)** represents the model where an intracellular compartment is introduced to sequester uptaken TYR as an alternative to assymetric facilitated diffusion transport.

Previous studies examining TYR membrane passage in different cellular systems, have confirmed that TYR can readily diffuse across synthetic lipid bilayers with a permeability coefficient (ρ) of 22.6 ± 4.3 Å/s (Berry et al., 2013), and *via* a facilitated diffusion transport process mediated by a transporter with the pharmacological characteristics of organic cation transporter 2 (OCT2; *Slc22A2*) in native neuronal preparations (K_t_ = 101.5 nM; V_max_ = 30.2 fmol/mg protein/s) (Berry et al., 2016). In Caco-2 intestinal epithelial cells, we have previously shown that TYR is also transported across the apical membrane by OCT2 (Sarkar and Berry, 2020). In contrast, TYR transport across the basolateral membrane involves an unidentified, Na^+^-dependent, active transporter (Figure 1), with a K_t_ = 33.1 nM and a V_max_ = 43.0 nM/s (Sarkar and Berry, 2020). The presence of such an active transport process could mask the presence of additional TYR transporters, particularly in the basolateral membrane. The current study has, therefore, used computer modelling to determine whether the known kinetics of the identified transporter systems are sufficient to explain the experimentally observed TYR concentration-time relationships in the Caco-2 experimental system (Sarkar and Berry, 2020), or whether additional transport factors are required.

## 2 Methods

### 2.1 Study model

For known TYR transport processes (Berry et al., 2013; Berry et al., 2016; Sarkar and Berry, 2020) ordinary differential equations (ODEs) were developed. Michaelis-Menten kinetics were assumed for all transporter-mediated processes, and the generalized equations (Friedman, 2008) for facilitated diffusion Eqn. 1 and active Eqn. 2 transporters used, where X_1_ represents the concentration of TYR in the donor compartment and X_2_ the concentration in the receiver compartment.

$$\frac{dX_{1}}{dt}=-\frac{V_{\max}X_{1}}{K_{t}+X_{1}}+\frac{V_{\max}X_{2}}{K_{t}+X_{2}}$$ (1)

$$\frac{dX_{1}}{dt}=-\frac{V_{\max}X_{1}}{K_{t}+X_{1}}$$ (2)

A one phase exponential decay function was used for simple (non-transporter mediated) diffusion (Friedman, 2008) (Eq. 3), where A represents the surface area of the membrane. This was assumed to be equal to the surface area of the transwell insert for a Caco-2 monolayer.

$$\frac{dX_{1}}{dt}=\frac{-\left(p\times A\right)}{Vol}\;\left(X_{1}-X_{2}\right)$$ (3)

These ODEs were used to develop a three-compartment mathematical model using MATLAB (R2021a) to predict the concentration-time relationship of TYR changes in each of the apical (Eq. 4), intracellular (Eq. 5), and basolateral compartments (Eq. 6) for both luminal-to-blood (apical loading) and blood-to-luminal (basolateral loading; see Figure 1A) transport. In all equations, blue text corresponds to simple diffusion, green text to OCT2 mediated facilitated diffusion transport, and purple text to the basolateral Na^+^-dependent active transporter. The α term represents a constant which is the product of ρ and A . Volume terms are incorporated in to equations representing active and facilitated transporters (e.g. Eqs 4–6) as the V_max_ values were determined for the cellular compartment. Since there is a large volume difference between the cellular compartment and the apical and basolateral compartments, in order to properly account for mass transfer, volume ratios needed to be included for the apical and basolateral compartments, which differ in volume from the cellular compartment since, for example, a molecular flux between compartments will cause a smaller change in concentration in the larger compartment compared to the smaller compartment. Vol_apical_ (= 100 μL) and Vol_baso_ (= 600 μL), represent the experimental volumes of the apical and basolateral compartments, respectively. Vol_cell_ (= 0.8168 μL) represents the previously calculated intracellular volume of the Caco-2 monolayer (Sarkar and Berry, 2020).

$$\frac{dX_{apical}}{dt}=\left[-\left(\frac{\alpha }{Vol_{apical}}\right)\times \left(X_{apical}-X_{cell}\right)\right]+\left[-\left(\frac{Vol_{cell}}{Vol_{apical}}\right)\times \left(\frac{V_{\max\_OCT2}X_{apical}}{K_{t\_OCT2}+X_{apical}}-\frac{V_{\max\_OCT2}X_{cell}}{K_{t\_OCT2}+X_{cell}}\right)\right]$$ (4)

$$\frac{dX_{cell}}{dt}=\left[-\left(\frac{\alpha }{Vol_{cell}}\right)\times \left(X_{cell}-X_{apical}\right)\right]+\left[-\left(\frac{\alpha }{Vol_{cell}}\right)\times \left(X_{cell}-X_{baso}\right)\right]+\left(\frac{V_{\max\_OCT2}X_{apical}}{K_{t\_OCT2}+X_{apical}}-\frac{V_{\max\_OCT2}X_{cell}}{K_{t\_OCT2}+X_{cell}}\right)+\left(\frac{V_{\max\_baso\_active\;}X_{baso}}{K_{t\_baso\_active}+X_{baso}}\right)$$ (5)

$$\frac{dX_{baso}}{dt}=\left[-\left(\frac{\alpha }{Vol_{baso}\;}\right)\times \left(X_{baso}-X_{cell}\right)\right]+\left[-\left(\frac{Vol_{cell}}{Vol_{baso}}\right)\times \left(\frac{V_{\max\_baso\_active}X_{baso}}{K_{t\_\_baso\_active}+X_{baso}}\right)\right]$$ (6)

The previously reported literature value for synaptosomal OCT2 V_max_ of 30.2 fmol/mg protein/s (Berry et al., 2016) was converted to nM/s (0.11 nM/s) using the known protein content that had previously been determined for each synaptosomal preparation. Starting concentrations in apical and basolateral compartments were set to those previously determined experimentally (Sarkar and Berry, 2020) and predicted concentration-time relationships in each compartment were compared to our previously published experimental observations (Sarkar and Berry, 2020).

### 2.2 Addition of facilitated diffusion transporters to the Caco-2 basolateral membrane

On the basis of initial modelling, an additional facilitated diffusion transporter (red text) was introduced to the basolateral membrane (Eqs 7 and 8; Figure 1B).

$$\begin{array}{l} \frac{dX_{cell}}{dt}=\left[-\left(\frac{\alpha }{Vol_{cell}}\right)\times \left(X_{cell}-X_{apical}\right)\right]+\left[-\left(\frac{\alpha }{Vol_{cell}}\right)\times \left(X_{cell}-X_{baso}\right)\right]+\\ \left(\frac{V_{\max\_OCT2}X_{apical}}{K_{t\_OCT}+X_{apical}}-\frac{V_{\max\_OCT2}X_{cell}}{K_{t\_OCT}+X_{cell}}\right)+\left(\frac{V_{\max\_baso\_active}X_{baso}}{K_{t\_baso\_active}+X_{baso}}\right)\\ +\left(\frac{V_{\max\_baso\_new\_FD}X_{baso}}{K_{t\_baso\_new\_FD}+X_{baso}}-\frac{V_{\max\_baso\_new\_FD}X_{cell}}{K_{t\_baso\_new\_FD}+X_{cell}}\right) \end{array}$$ (7)

$$\frac{dX_{baso}}{dt}=\left[-\left(\frac{\alpha }{Vol_{baso}\;}\right)\times \left(X_{baso}-X_{cell}\right)\right]+\left[-\left(\frac{Vol_{cell}}{Vol_{baso}}\right)\times \left(\frac{V_{\max\_baso\_active}X_{baso}}{K_{t\_baso\_active}+X_{baso}}\right)\right]+\left[-\left(\frac{Vol_{cell}}{Vol_{baso}}\right)\times \left(\frac{V_{\max\_baso\_new\_FD}X_{baso}}{K_{t\_baso\_new\_FD}+X_{baso}}-\frac{V_{\max\_baso\_new\_FD}X_{cell}}{K_{t\_baso\_new\_FD}+X_{cell}}\right)\right]$$ (8)

Initially, kinetic parameters were set to those of OCT2, as this transporter is already identified as contributing to TYR transport in Caco-2 cells (Sarkar and Berry, 2020). Since OCT2 is present in the apical membrane (Sarkar and Berry, 2020), we reasoned that its presence in the basolateral membrane could also be possible. To test for putative density differences in OCT2 between the apical and basolateral membranes, the V_max_baso_new_FD_ term was allowed to vary from the experimentally determined V_max_OCT2_ term in the apical membrane. V_max_baso_new_FD_ was varied from 0.1x to 20x the apical membrane V_max_ of OCT2. To determine if a basolateral membrane facilitated diffusion transporter distinct from OCT2 improved consistency with experimental observations, both V_max_baso_new_FD_ and K_t_baso_new_FD_ were systematically varied (Eqs 7 and 8), red text).

### 2.3 Incorporation of asymmetry in facilitated diffusion transporters

Results from the modelling described in section 2.2 indicated that asymmetry of TYR facilitated diffusion transporters may be required in order to match experimental observations. To attain this, distinct K_t_ values were introduced for OCT2 transport in the apical to cell (K_t_OCT2_apicaltocell_) and cell to apical (K_t_OCT2_celltoapical_) directions (Eqs 9 and 10). Similarly, different K_t_ values for the basolateral to cell (K_t_baso_new_FD_basotocell_) and cell to basolateral (K_t_baso_new_FD_celltobaso_) directions of the added basolateral membrane facilitated diffusion transporter were introduced (Eqs 10 and 11).

$$\frac{dX_{apical}}{dt}=\left(\frac{\alpha }{Vol_{apical}\;}\right)\times \left(X_{cell}-X_{apical}\right)+\left(\frac{Vol_{cell}}{Vol_{apical}}\right)\times \left(\frac{V_{\max\_OCT2}X_{cell}}{K_{t\_OCT2\_celltoapical}+X_{cell}}-\frac{V_{\max\_OCT2}X_{apical}}{K_{t\_OCT2\_apicaltocell}+X_{apical}}\right)$$ (9)

$$\begin{array}{l} \frac{dX_{cell}}{dt}=[\left(\frac{\alpha }{Vol_{cell}\;}\right)\times \left(X_{apical}-X_{cell}\right)]+\left[\left(\frac{\alpha }{Vol_{cell}}\right)\times \left(X_{baso}-X_{cell}\right)\right]\\ +\left(\frac{V_{\max\_OCT2}X_{apical}}{K_{t\_OCT2\_apicaltocell}+X_{apical}}-\frac{V_{\max\_OCT2}X_{cell}}{K_{t\_OCT2\_celltoapical}+X_{cell}}\right)+\left(\frac{V_{\max\_baso\_active}X_{baso}}{K_{t\_baso\_active}+X_{baso}}\right)+\left(\frac{V_{\max\_baso\_new\_FD}X_{baso}}{K_{t\_baso\_new\_FD\_basotocell}+X_{baso}}-\frac{V_{\max\_baso\_new\_FD}X_{cell}}{K_{t\_baso\_new\_FD\_celltobaso}+X_{cell}}\right) \end{array}$$ (10)

$$\begin{array}{c} \frac{dX_{baso}}{dt}=\left[-\left(\frac{\alpha }{Vol_{baso}\;}\right)\times \left(X_{baso}-X_{cell}\right)\right]+\\ \left[-\left(\frac{Vol_{cell}}{Vol_{baso}}\right)\times \left(\frac{V_{\max\_baso\_active}X_{baso}}{K_{t\_baso\_active}+X_{baso}}\right)\right]+\\ \left[-\left(\frac{Vol_{cell}}{Vol_{baso}}\right)\times \left(\frac{V_{\max\_baso\_new\_FD}X_{baso}}{K_{t\_baso\_new\_FD\_basotocell}+X_{baso}}-\frac{V_{\max\_baso\_new\_FD}X_{cell}}{K_{t\_baso\_new\_FD\_celltobaso}+X_{cell}}\right)\right] \end{array}$$ (11)

### 2.4 Introduction of TYR intracellular compartmentalization

To model for potential intracellular compartmentalization of TYR, a compartmentalization factor, ‘z’, was introduced to restrict the amount of intracellular TYR that was available for transport out of Caco-2 cells (Eqs 12–14), where 0 ≤ z ≤ 1.

$$\begin{array}{l} \frac{dX_{apical}}{dt}=\left[-\left(\frac{\alpha }{Vol_{apical}}\right)\times \left(X_{apical}-z*X_{cell}\right)\right]\\ +\left[-\left(\frac{Vol_{cell}}{Vol_{apical}}\right)\times V_{\max\_OCT2}\left(\frac{X_{apical}}{K_{t\_OCT2\_apicaltocell}+X_{apical}}-\frac{z*X_{cell}}{K_{t\_OCT2\_celltoapical}+z*X_{cell}}\right)\right] \end{array}$$ (12)

$$\begin{array}{l} \frac{dX_{cell}}{dt}=\left[-\left(\frac{\alpha }{Vol_{cell}}\right)\times \left(z*X_{cell}-X_{apical}\right)\right]+\left[-\left(\frac{\alpha }{Vol_{cell}}\right)\times \left(z*X_{cell}-X_{baso}\right)\right]+\\ V_{\max\_OCT2}\left(\frac{X_{apical}}{K_{t\_OCT2\_apicaltocell}+X_{apical}}-\frac{z*X_{cell}}{K_{t\_OCT2\_celltoapical}+z*X_{cell}}\right)\\ +\left(\frac{V_{\max\_baso\_active}}{K_{t\_baso\_active}+X_{baso}}\right)\\ +V_{\max\_baso\_new\_FD}\left(\frac{X_{baso}}{K_{t\_baso\_new\_FD\_basotocell}+X_{baso}}-\frac{z*X_{cell}}{K_{t\_baso\_new\_FD\_celltobaso}+z*X_{cell}}\right) \end{array}$$ (13)

$$\begin{array}{l} \frac{dX_{baso}}{dt}=\left[-\left(\frac{\alpha }{Vol_{baso}\;}\right)\times \left(X_{baso}-z*X_{cell}\right)\right]+\\ \left[-\left(\frac{Vol_{cell}}{Vol_{baso}}\right)\times \left(\frac{V_{\max\_bas\_\_active}}{K_{t\_baso\_active}+X_{baso}}\right)\right]+\\ {}[-\left(\frac{Vol_{cell}}{Vol_{baso}}\right)\times V_{\max\_baso\_new\_FD}\left(\frac{X_{baso}}{K_{t\_baso\_new\_FD\_basotocell}+X_{baso}}-\frac{z*X_{cell}}{K_{t\_baso\_new\_FD\_celltobaso}+z*X_{cell}}\right)] \end{array}$$ (14)

To provide a more refined description of such potential compartmentalization, Michaelis-Menten terms (brown text) for the transport of TYR into an inner compartment were also introduced (Eqs 15–18); Figure 1C), and the volume of the intracellular compartment (Vol_inner_) set at 10% of the total volume of the cell, to estimate a likely upper boundary for an intracellular compartment.

$$\frac{dX_{apical}}{dt}=-\left[\left(\frac{\alpha }{Vol_{apical}}\right)\times \left(X_{apical}-X_{cell}\right)+\left(\frac{Vol_{cell}}{Vol_{apical}}\right)\times V_{\max\_OCT2}\left(\frac{X_{apical}}{K_{t\_OCT2\_apicaltocell}+X_{apical}}-\frac{X_{cell}}{K_{t\_OCT2\_celltoapical}+X_{cell}}\right)\right]$$ (15)

$$\begin{array}{l} \frac{dX_{cell}}{dt}=\left[-\left(\frac{\alpha }{Vol_{cell}}\right)\times \left(X_{cell}-X_{apical}\right)\right]+\left[-\left(\frac{\alpha }{Vol_{cell}}\right)\times \left(X_{cell}-X_{baso}\right)\right]+V_{\max\_OCT2}\left(\frac{X_{apical}}{K_{t\;\_OCT2\_apicaltocell\;}+X_{apical}}-\frac{X_{\_cell}}{K_{t\;\_OCT2\_celltoapical\;}+X_{\_cell}}\right)\\ +\left(\frac{V_{\max\_baso\_active\_transporter\;}X_{baso}}{K_{t\_baso\_active\_transporter\;}+X_{baso}}\right)+V_{\max\_baso\_new\_FD\_transporter\;}\left(\frac{X_{baso}}{K_{t\_baso\_new\_FD\_basotocell\_transporter}+X_{baso}}-\frac{X_{\_cell}}{K_{t\_baso\_new\_FD\_celltobaso\_transporter\;}+X_{\_cell}}\right)-\left(\frac{V_{\max\_i}X_{cell}}{K_{t\_i}+X_{cell}}\right) \end{array}$$ (16)

$$\begin{array}{l} \frac{dX_{baso}}{dt}=\left[-\left(\frac{\alpha }{Vol_{baso}\;}\right)\times \left(X_{baso}-X_{\_cell}\right)\right]\\ +\left[-\left(\frac{Vol_{original\_cell}}{Vol_{baso}}\right)\times \left(\frac{V_{\max\_baso\_AT}X_{baso}}{K_{baso\_AT}+X_{baso}}\right)\right]\\ +\left[-\left(\frac{Vol_{cell\_1}}{Vol_{baso}}\right)\times V_{\max\_baso\_FD\_new}\left(\frac{X_{baso}}{K_{t\_baso\_FD\_new\_baso}+X_{baso}}-\frac{X_{\_cell}}{K_{t\_baso\_FD\_new\_cell}+X_{\_cell}}\right)\right] \end{array}$$ (17)

$$\frac{dX_{i}}{dt}=\left(\frac{Vol_{cell}}{Vol_{inner}}\right)\times \left(\frac{V_{\max\_i}X_{cell}}{K_{t\_i}+X_{cell}}\right)$$ (18)

### 2.5 Least squares determination of the kinetic parameters of unknown TYR transporter kinetics.

In order to estimate transporter parameters from experimental data, e.g., the various V_max_ and K_t_ values on the right-hand sides (RHSs) of Eqs 9–11, two least-squares fitting procedures were employed. The first involved comparing experimental values for the rate of change of the concentration for a compartment, e.g., the left-hand sides of Eqs 9–11, with model predictions (the RHSs). The second procedure involved solving the ODEs at each iteration of the fitting procedure and comparing model and experimental concentrations. As the fits were non-linear with several parameters, choices for the initial values of the parameters for the fitting procedures were informed by considering constraints on parameter values implied by the model, given the experimental data, reducing the need for exhaustive searches over initial parameter values. Some of these fitting calculations were carried using Mathematica (v12.1.1).

We first describe the fitting procedure for determining model parameters by considering the rate of change of the concentration. As an illustrative example, we consider fitting V_max_OCT2_ and K_t_OCT2_ in the model comprising Eqs 4–6. Note that compartmental volumes and α are given and are therefore not fit parameters.

Step 1 Linear fit of the experimental data

Linear regression analysis was performed on previously published (Sarkar and Berry, 2020) concentration-time relationships in order to determine the rate of change of TYR concentration in each of the apical, cellular, and basolateral compartments following both apical and basolateral compartment TYR loading. In all six cases, the data were well described by constant rates of change (concentrations changing linearly in time). Thus, all the experimental data are described by six “slopes”.

Step 2 Development of objective functions

Eqs 4–6 were rearranged (Eqs 19–21 respectively) so that all experimentally known quantities including the determined rate, appear on the right-hand side (RHS). Terms with parameters to be solved appear on the left-hand side (LHS).

$$-\left(\frac{Vol_{cell}}{Vol_{apical}}\right)\times \left(\frac{V_{\max\_OCT2}X_{apical}}{K_{t\_OCT2}+X_{apical}}-\frac{V_{\max\_OCT2}X_{cell}}{K_{t\_OCT2}+X_{cell}}\right)=\frac{dX_{apical}}{dt}+\left(\frac{\alpha }{Vol_{apical}}\right)\times \left(X_{apical}-X_{cell}\right)$$ (19)

$$\frac{V_{\max\_OCT2}X_{apical}}{K_{t\_OCT2}+X_{apical}}-\frac{V_{\max\_OCT2}X_{cell}}{K_{t_{OCT2}}+X_{cell}}+\frac{V_{\max\_baso\_active\;}X_{baso}}{K_{t_{baso_{active}}}+X_{baso}}=\frac{dX_{cell}}{dt}+\left(\frac{\alpha }{Vol_{cell}}\right)\times \left(X_{cell}-X_{apical}\right)+\left(\frac{\alpha }{Vol_{cell}}\right)\times \left(X_{cell}-X_{baso}\right)$$ (20)

$$-\left(\frac{Vol_{cell}}{Vol_{baso}}\right)\times \left(\frac{V_{\max\_baso\_active\;}X_{baso}}{K_{t_{baso_{active}}}+X_{baso}}\right)=\frac{dX_{baso}}{dt}+\left(\frac{\alpha }{Vol_{baso}\;}\right)\times \left(X_{baso}-X_{cell}\right)$$ (21)

This rearrangement suggests using the LHSs to define fitting functions F_apical_, F_cell_ and F_baso_ (Eqs 22–24),

$$F_{apical}\left(V_{\max\_OCT2},K_{t\_OCT2};X_{apical},X_{cell}\right)=-\left(\frac{Vol_{cell}}{Vol_{apical}}\right)\times \left(\frac{V_{\max\_OCT2}X_{apical}}{K_{t\_OCT2}+X_{apical}}-\frac{V_{\max\_OCT2}X_{cell}}{K_{t\_OCT2}+X_{cell}}\right),$$ (22)

$$F_{cell}\left(V_{\max\_OCT2},K_{t\_OCT2};X_{apical},X_{cell},X_{baso}\right)=\left(\frac{V_{\max\_OCT2}X_{apical}}{K_{t\_OCT2}+X_{apical}}-\frac{V_{\max\_OCT}X_{cell}}{K_{t\_OCT2}+X_{cell}}\right)+\frac{V_{\max\_baso\_active\;}X_{baso}}{K_{t\_baso\_active}+X_{baso}},$$ (23)

$$F_{baso}\left(V_{\max\_OC},K_{t\_OCT2};X_{baso}\right)=-\left(\frac{Vol_{cell}}{Vol_{baso}}\right)\times \left(\frac{V_{\max\_baso\_active\;}X_{baso}}{K_{t\_baso\_active}+X_{baso}}\right).$$ (24)

The compartmental concentrations were determined experimentally from t = 0 to t = 30 min at intervals of 5 min, and thus these three functions can be written as functions of time, e.g. Eq. 25,

$$\begin{array}{l} F_{apical}\left(V_{\max\_OCT2},K_{t\_OCT2};t\right)\\ =F_{apical}\left(V_{\max\_OCT2},K_{t\_OCT};X_{apical}\left(t\right),X_{cell}\left(t\right)\right). \end{array}$$ (25)

We also use the RHSs of equations 19)–21) to define known, time-dependent quantities f_apical_(t), f_cell_(t) and f_baso_(t), respectively. The ‘f’ is a term that contains small diffusive terms and derivatives of TYR concentration of TYR with respect to time (dX/dt). The TYR concentration data in all the compartments have been fit by linear regression. In that case, all of the dX/dt terms in the ‘f’ terms become constant and, therefore, the ‘f’ terms have only a very weak time dependence because of the very small diffusive terms

Objective functions (Q) were then defined for each compartment as shown in Eqs 26–28 and represent the squared differences between F and f calculated at each time point t.

$$Q_{apical}=\overset{T}{\underset{t=0}{\sum }}{\left(F_{apical}\left(V_{\max\_OCT2},K_{t\_OCT2};t\right)-f_{apical}\left(t\right)\right)}^{2}$$ (26)

$$Q_{cell}=\overset{T}{\underset{t=0}{\sum }}{\left(F_{cell}\left(V_{\max\_OCT2},K_{t\_OCT2};t\right)-f_{cell}\left(t\right)\right)}^{2}$$ (27)

$$Q_{baso}=\overset{T}{\underset{t=0}{\sum }}{\left(F_{baso}\left(V_{\max\_OCT2},K_{t\_OCT2};t\right)-f_{baso}\left(t\right)\right)}^{2}$$ (28)

Global objective functions were then written for apical ( $Q_{A})$ and basolateral $(Q_{B}$ ) loading as the sum of the individual compartment objective functions following apical or basolateral loading respectively (Eqs 29 and 30).

$$Q_{A}=Q_{apical,apical\_addition}+Q_{cell,apical\_addition}+Q_{baso,apical\_addition}$$ (29)

$$Q_{B}=Q_{apical,baso\_addition}+Q_{cell,baso\_addition}+Q_{baso,baso\_addition}$$ (30)

Q_A_ and Q_B_ can then be minimized simultaneously by defining an overall function Q (Eqs 31), providing a least squares minimized solution for the individual kinetic terms in Eqs 19–21

$$Q=Q_{A}+Q_{B}$$ (31)

Step3 Objective functions based on concentrations

In this fitting procedure, which we used to complement and validate the first one, the F functions in Eqs 26–28 were redefined to be the solutions to Eqs 19–21. For example, F_apical_ (V_max_OCT2_, K_t_OCT2_; t) = X_apical_(t), where X_apical_(t) comes from the numerical solution for Eqs 19–21. The quantities f in Eqs 26–28 were simply redefined to be the compartmental concentrations determined from experiment e.g. f_apical_ = X_apical_(t), where X_apical_(t) was determined from experiment. As the cellular concentrations were generally larger than in the other two compartments, we reduced both Q_cell,apical_ and Q_cell,basolateral_ in Q_A_ and Q_B_, respectively, by a factor *w*. The value of *w* affects the results, and we found that a value of 1,000 worked well. The dependence of the fitting procedure on *w* was mitigated by choosing good initial parameter values, as described in Step 4.

Step 4 Introduction of model order reduction

Non-linear fitting with many fit parameters can be difficult: there are in general many local minima of the objective function in parameter space, meaning similar fits can be achieved with very different parameter values. Results depend on the initial parameter values chosen when minimizing the objective function. In this final step, approximations were introduced to decrease the number of parameters that needed to be solved simultaneously, thereby simplifying the computational complexity. This allowed us to interactively explore ranges of parameter values and thus determine reasonable starting values. The procedure was based on approximating experimental concentrations as constant values or as being linear in time and on simplifying the model ODEs. For example, for the model represented by Eqs 12–14, $K_{t\_OCT2\_celltoapical}$ was approximated as follows.

Considering Eq. 12, we assumed that simple diffusion was negligible, α≈0, which allowed Eq. 12 to be simplified to Eq. 32,

$$\frac{dX_{apical}}{dt}≅\left[-\left(\frac{Vol_{cell}}{Vol_{apical}}\right)\times V_{\max\_OCT2}\left(\frac{X_{apical}}{K_{t_{OCT2_{apicaltocell}}}+X_{apical}}-\frac{z*X_{cell}}{K_{t_{OCT2_{celltoapical}}}+z*X_{cell}}\right)\right].$$ (32)

For basolateral addition at timepoint t = 0, we then substituted for the following experimental parameters: at t = 0, $X_{apical}=0,X_{cell}\approx 500$ nM (refer to Figure 4B) and dX_apical_/dt = 0.3168 nM/min = 0.00528 nM/s (the slope of the linear fit of the experimental data). This allows Eq. 32 to be reduced to Eq. 33.

$$0.00528≅\left[0.0082V_{\max\_OCT2}\left(\frac{500z}{K_{t\_OCT2\_celltoapical}+500z}\right)\right]$$ (33)

Rearranging to Eq. 34 allows $K_{t\_OCT2\_celltoapical}$ to be expressed as a function of other variables. For solutions where no intracellular compartment was modelled z was set to 1.

$$K_{t\_OCT2\_celltoapical}=\left(-500+768.65V_{\max\_OCT2}\right)z$$ (34)

Similar approximation techniques were also used for expressing:

1) $K_{t\_OCT2\_apicaltocell}$ in relation to $V_{\max\_OCT2}$ and z
2) $K_{t\_baso\_new\_FD\_celltobaso}$ in relation to $V_{\max\_baso\_new\_FD}$

3) $K_{t\_baso\_active\_transporter}$ in relation to $V_{\max\_OCT2}$ , $V_{\max\_baso\_new\_FD\_transporter}$ , $K_{t\_baso\_new\_FD\_basotocell}$ and z

Such parameter constraints reduce the number of fitting parameters and simplified choosing initial parameter value sets that already described the data fairly well. We did not use the constraints when minimizing the objective function because of the approximate nature of the constraints.

## 3 Results

### 3.1 Known TYR transporters do not recapitulate experimental observations

**FIGURE 2:**
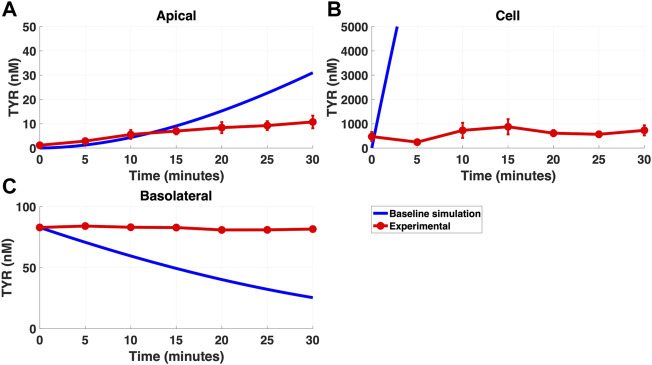
The baseline model does not recapitulate experimental data for TYR transport following basolateral loading. The transport of 82.9 nM TYR across Caco-2 cells was modeled for basolateral loading by solving Eqs 4–6, using the values mentioned in Supplementary Table S2, using MATLAB vR 2021a. Comparisons were made between the baseline model (representing known TYR transporters that have been characterized; blue curve) for the apical Panel 2**(A)**, cellular Panel 2**(B)** and basolateral compartments Panel 2**(C)**, *versus* experimental observations; red curves (Sarkar and Berry, 2020). Kinetic parameters used for this modelling were: V_max_OCT2_ = 0.1 nM/s, K_t _OCT2_apicaltocell_ = 101.5 nM, V_max_baso_active_ = 43.0 nM/s and K_t_baso_active_ = 33.1 nM.

**FIGURE 3:**
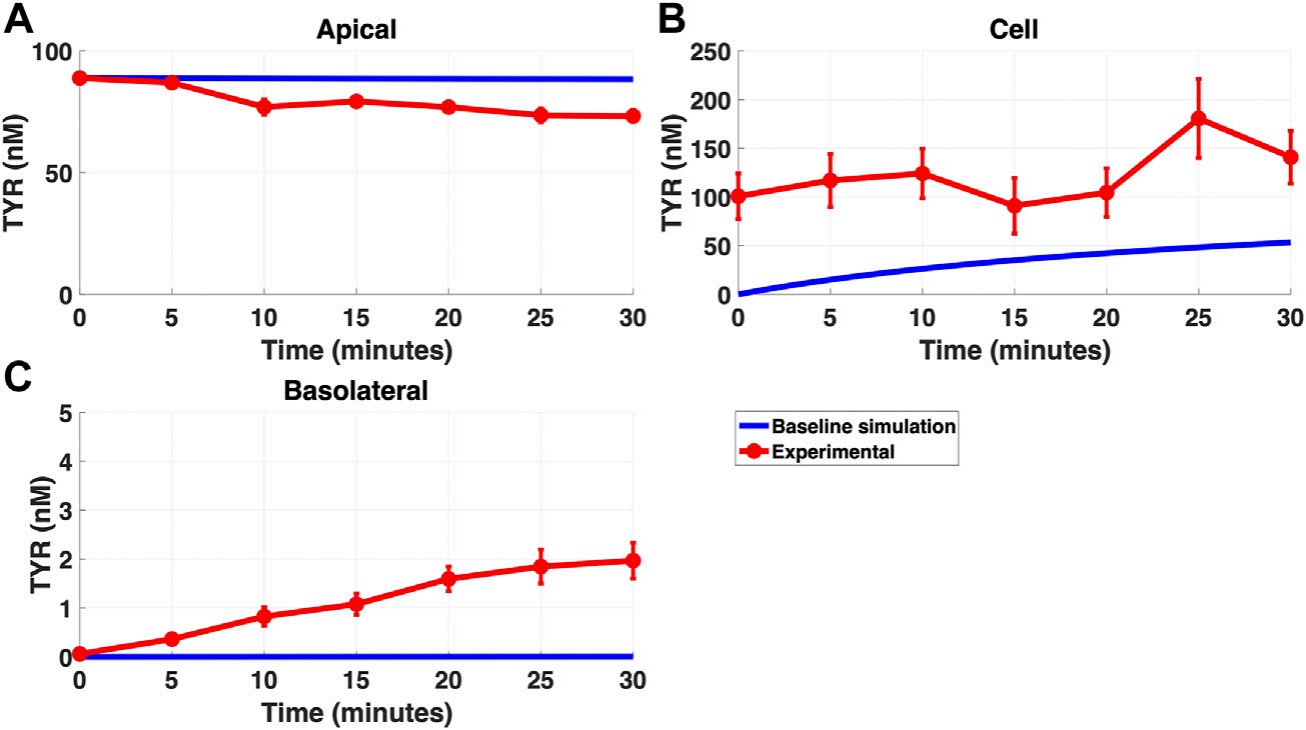
The baseline model does not fully recapitulate experimental data for TYR transport following apical loading. The transport of 88.9 nM TYR across Caco-2 cells was modeled for apical loading by solving Eqs 4–6, using the values mentioned in Supplementary Table S2, using MATLAB vR 2021a. Comparisons were made between the baseline model (representing known TYR transporters been characterized; blue curve) for the apical Figure 3**(A)**, cellular Figure 3**(B)** and basolateral compartments Figure 3**(C)**, *versus* experimental observations; red curves (Sarkar and Berry, 2020). Kinetic parameters used for this modelling were: V_max_OCT2_ = 0.1 nM/s, K_t _OCT2_apicaltocell_ = 101.5 nM, V_max_baso_active_ = 43.0 nM/s and K_t_baso_active_ = 33.1 nM.

Modelling TYR transport across a Caco-2 cell barrier following basolateral compartment loading, using the kinetics of previously identified transporter systems, was not able to recapitulate experimental observations (Figures 2A–C). Conversely, following apical compartment loading, restricting the model to known transporter kinetics partially matched experimental observations for apical to cellular transport (Figures 3A,B), but not for cellular to basolateral transport (Figures 3B,C).

### 3.2 Addition of a basolateral membrane facilitated diffusion transporter does not improve modelling

**FIGURE 4:**
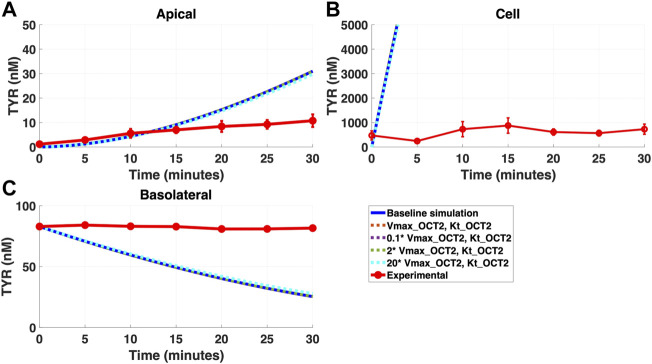
Introduction of OCT2, at a range of densities, to the Caco-2 basolateral membrane does not improve model accuracy for basolateral TYR loading. The transport of 82.9 nM of TYR across Caco-2 cells was modeled following basolateral loading by solving Eqs 4–8, using the values mentioned in Supplementary Table S2, using MATLAB vR 2021a. Comparisons were made between the models (baseline = blue curve; modified = dotted curves) for the apical Panel 4**(A)**, cellular Panel 4**(B)** and basolateral compartments Panel 4**(C)**, *versus* experimental observations; red curve (Sarkar and Berry, 2020). Addition of an OCT2-like transporter to the basolateral membrane at varying densities did not significantly alter modelled TYR concentration-time profiles. All of the curves have overlapped except experimental curve. Kinetic parameters used for this modelling were: V_max_OCT2_ = 0.1 nM/s, K_t _OCT2_apicaltocell_ = 101.5 nM, V_max_baso_active_ = 43.0 nM/s, K_t_baso_active_ = 33.1 nM, V_max_baso_new_FD_ = 0.01–2 nM/s and K_t_baso_new_FD_basotocell_ = 101.5 nM.

**FIGURE 5:**
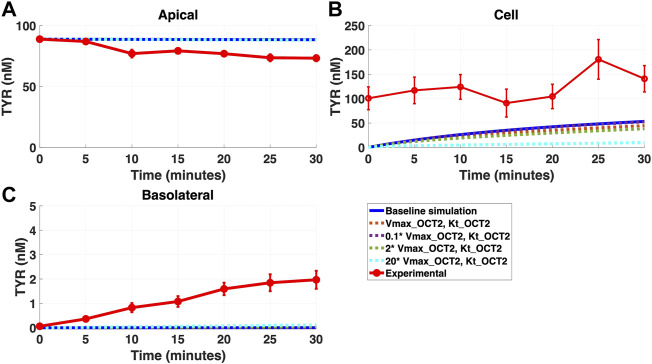
Introduction of OCT2 at variable densities to the Caco-2 basolateral membrane worsens model accuracy following apical TYR loading. The transport of 88.9 nM TYR across Caco-2 cells was modeled following basolateral loading by solving Eqs 4–8, using the values mentioned in Supplementary Table S2, using MATLAB vR 2021a. Comparisons were made between the models (baseline = blue curve; modified = dotted curves) for the apical Panel 5**(A)**, cellular Panel 5**(B)** and basolateral compartments Panel 5**(C)**, *versus* experimental observations; red curve (Sarkar and Berry, 2020). Addition of an OCT2-like transporter to the basolateral membrane at varying densities either had no effect, or worsened, modelling of TYR concentration-time profiles. Some of the curves have overlapped except experimental curve. Kinetic parameters used for this modelling were: V_max_OCT2_ = 0.1 nM/s, K_t _OCT2_apicaltocell_ = 101.5 nM, V_max_baso_active_ = 43.0 nM/s, K_t_baso_active_ = 33.1 nM, V_max_baso_new_FD_ = 0.01–2 nM/s and K_t_baso_new_FD_basotocell_ = 101.5 nM.

Addition of OCT2 to the basolateral membrane at densities ranging from 0.1-20X that observed in the apical membrane, did not improve model accuracy following either basolateral (Figures 4A–C) or apical (Figures 5A–C) compartment loading with TYR.

**FIGURE 6:**
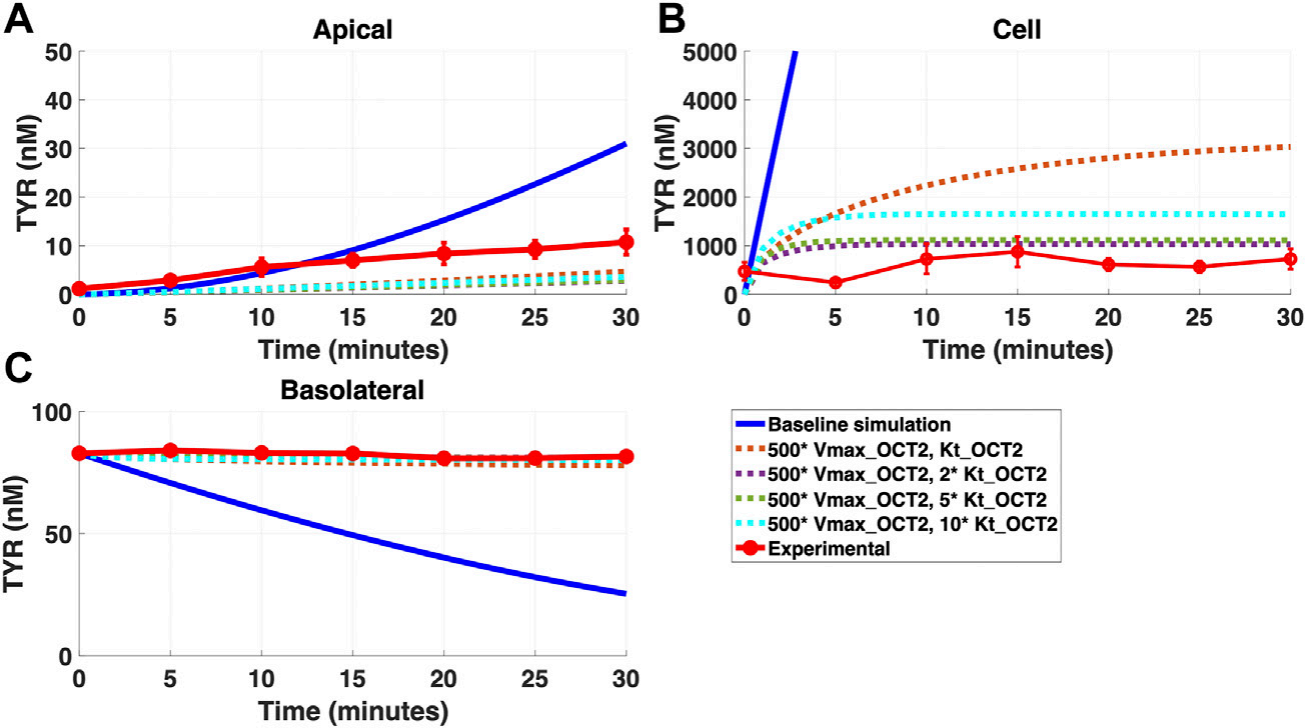
Introduction of a non-OCT2 facilitated diffusion transporter to the Caco-2 basolateral membrane improves model accuracy following basolateral TYR loading. The transport of 82.9 nM TYR across Caco-2 cells was modeled following basolateral loading by solving Eqs 4–8, using the values mentioned in Supplementary Table S2, using MATLAB vR 2021a. Comparisons were made between the models (baseline = blue curve; modified = dotted curves) and experimental observations; red curve (Sarkar and Berry, 2020)for the apical Panel 6**(A)**, cellular Panel 6**(B)** and basolateral compartments Panel 6**(C)**. Good agreement with experimental observations were obtained at V_max_ = 50 nM/s (500*V_max___OCT2_) and K_t_ = 507.5–1,015 nM (5–10 *K_t_OCT2_). Kinetic parameters used for this modelling were: V_max_OCT2_ = 0.1 nM/s, K_t _OCT2_apicaltocell_ = 101.5 nM, V_max_baso_active_ = 43.0 nM/s, K_t_baso_active_ = 33.1 nM, V_max_baso_new_FD_ = 50 nM/s and K_t_baso_new_FD_basotocell_ = 101.5–1,015 nM.

**FIGURE 7:**
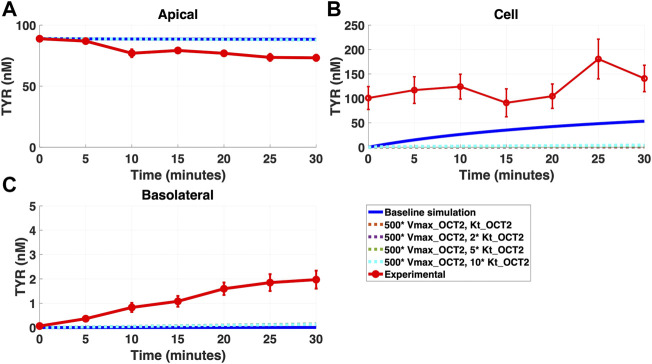
Introduction of a non-OCT2 facilitated diffusion transporter to basolateral membranes worsens model matching to experimental data following apical TYR loading. The transport of 88.9 nM of TYR across Caco-2 cells was modeled for apical loading by solving Eqs 4–8, using the values mentioned in Supplementary Table S2, using MATLAB vR 2021a. Comparisons were made between the models (baseline = blue curve; modified = dotted curves) and experimental observations; red curve (Sarkar and Berry, 2020) for the apical Panel 7**(A)**, cellular Panel 7**(B)** and basolateral compartments Panel 7**(C)**. Addition of the additional transporter to the basolateral membrane decreased the ability to model experimental data. Kinetic parameters used for this modelling were: V_max_OCT2_ = 0.1 nM/s, K_t _OCT2_apicaltocell_ = 101.5 nM, V_max_baso_active_ = 43.0 nM/s, K_t_baso_active_ = 33.1 nM, V_max_baso_new_FD_ = 50 nM/s and K_t_baso_new_FD_basotocell_ = 101.5–1,015 nM.

In contrast, addition of a facilitated diffusion transporter not confined to OCT2 K_t_ provided a closer match to experimental results following basolateral loading (Figures 6A–C) when kinetic parameters of V_max_ = 55 nM/s and K_t_ = 203–507.5 nM were utilized. These kinetic parameters, however, worsened the matching to experimental data following apical loading (Figures 7A–C).

### 3.3 Asymmetric apical membrane TYR transport by OCT2 allows recapitulation of experimental findings

**FIGURE 8:**
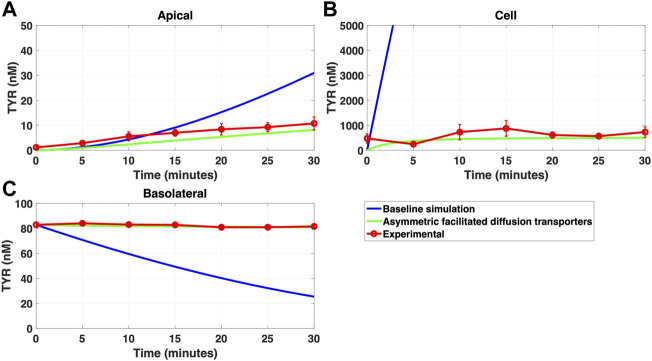
Asymmetry of OCT2 mediated transport recapitulates experimental observations following basolateral TYR loading. The transport of 82.9 nM TYR across Caco-2 cells was modeled following basolateral loading by solving Eqs 9–11, using the values mentioned in Supplementary Table S2, using MATLAB vR 2021a. Comparisons were made between the models (baseline = blue curve; modified = green curves) and experimental observations; red curve (Sarkar and Berry, 2020) for the apical Panel 8**(A)**, cellular Panel 8**(B)** and basolateral compartments Panel 8**(C)**. Asymmetric OCT2 transport combined with a fully bidirectional basolateral membrane facilitated diffusion transporter allowed experimental data to be modelled. Solved kinetic parameters from this modelling were: V_max_OCT2_ = 2.3 nM/s, K_t _OCT2_apicaltocell_ = 110.4 nM, K_t _OCT2_celltoapical_ = 1,227.9 nM, V_max_baso_active_ = 3.3 nM/s, K_t_baso_active_ = 29.0 nM, V_max_baso_new_FD_ = 6.0 nM/s, K_t_baso_new_FD_basotocell_ = 584.1 nM and K_t_baso_new_FD_celltobaso_ = 672.4 nM.

**FIGURE 9:**
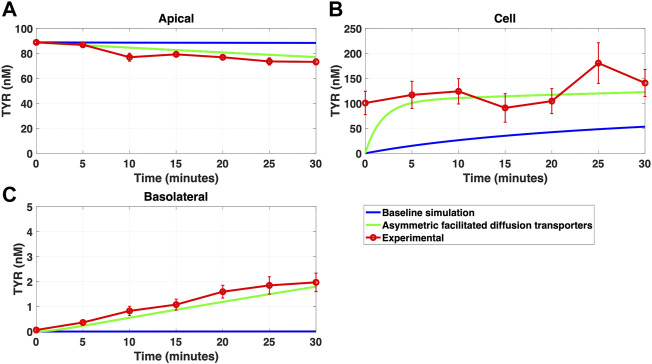
Introduction of asymmetry in the apical membrane OCT2 successfully recapitulates experimental observations following apical TYR loading. The transport of 88.9 nM of TYR across Caco-2 cells was modeled following apical loading by solving Eqs 9–11, using the values mentioned in Supplementary Table S2, using MATLAB vR 2021a. Comparisons were made between the models (baseline = blue curve; modified = green curves) and experimental observations; red curve (Sarkar and Berry, 2020) for the apical Panel 9**(A)**, cellular (Panel 9**(B)** and basolateral compartments (Panel 9**(C)**. The model reproduced experimental findings when asymmetric OCT2 transport was combined with a fully bidirectional basolateral membrane facilitated diffusion transporter. Solved kinetic parameters from this modelling were: V_max_OCT2_ = 2.3 nM/s, K_t _OCT2_apicaltocell_ = 110.4 nM, K_t _OCT2_celltoapical_ = 1,227.9 nM, V_max_baso_active_ = 3.3 nM/s, K_t_baso_active_ = 29.0 nM, V_max_baso_new_FD_ = 6.0 nM/s, K_t_baso_new_FD_basotocell_ = 584.1 nM and K_t_baso_new_FD_celltobaso_ = 672.4 nM.

Since improvement in model accuracy for basolateral loading came at a cost of worsening the accuracy for apical loading, we reasoned that one or more of the facilitated diffusion transporters needed to show asymmetric transport characteristics. Introducing such asymmetry allowed experimental concentration-time relationships to be recapitulated (Figures 8A–C and 9A–C) when an approximate 10-fold asymmetry in OCT2 was present in combination with symmetric transport by the added basolateral membrane facilitated diffusion transporter (Table 1).

**TABLE 1:** Required facilitated diffusion transporter kinetics for modelling of experimentally determined concentration-time relationships of TYR in each compartment.

Parameter	Predicted kinetics from model	Experimentally determined kinetics
V_max_OCT2_	2.3 nM/s	0.1 nM/s
K_t _OCT2_apicaltocell_	110.4 nM	101.5 nM
K_t _OCT2_celltoapical_	1,227.9 nM	101.5 nM
V_max_baso_active_	3.3 nM/s	43.0 nM/s
K_t_baso_active_	29.0 nM	33.1 nM
V_max_baso_new_FD_	6.0 nM/s	n/a
K_t_baso_new_FD_basotocell_	584.1 nM^*^	n/a
K_t_baso_new_FD_celltobaso_	672.4 nM^*^	n/a

Kinetic parameters of OCT2 and the added basolateral membrane transporter necessary to reproduce experimental concentration-time relationships in each compartment following either apical or basolateral TYR loading were solved for by least squares minimization following the development of objective functions to describe trans-cellular transport in each direction. *K_t_baso_new_FD_basotocell_ and K_t_baso_new_FD_celltobaso_ did not show a meaningful directional preference and so for future plots, the average value of 628.3 nM was used as the K_t_ for the basolateral bidirectional facilitated diffusion transporter.

### 3.4 Compartmentalization partially, but not fully, replaces the need for OCT2 asymmetry

**FIGURE 10:**
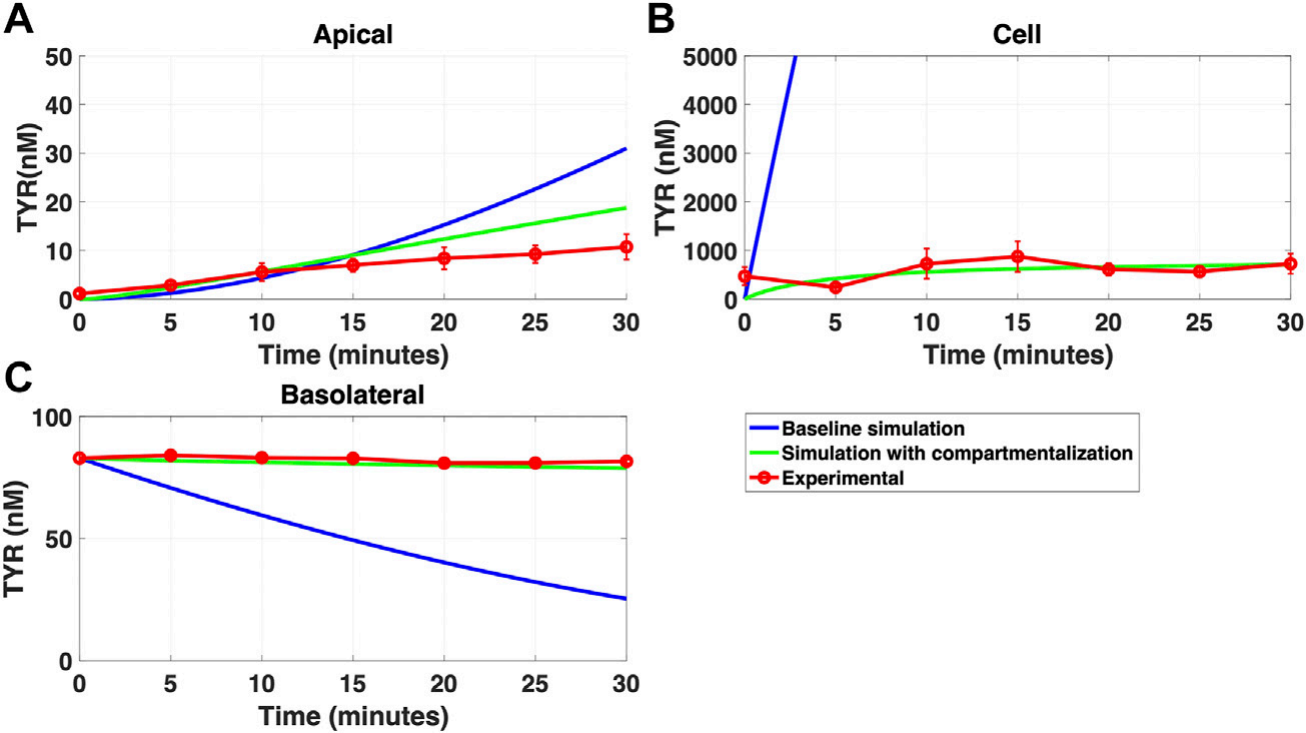
Compartmentalization is needed with introduction of symmetry in the facilitated diffusion transporters with basolateral TYR loading. The transport of 82.9 nM TYR across Caco-2 cells was modeled following basolateral loading by solving Eqs 12–14, using the values mentioned in Supplementary Table S2, using MATLAB vR 2021a. Comparisons were made between the models (baseline = blue curve; modified = green curves) and experimental observations; red curve (Sarkar and Berry, 2020) for the apical Panel 10**(A)**, cellular Panel 10**(B)** and basolateral compartments Panel 10**(C)**. When symmetry is introduced in the facilitated diffusion transporters, compartmentalization is needed. Kinetic parameters used for this modelling were: V_max_OCT2_ = 2.3 nM/s, K_t _OCT2_apicaltocell_ = 110.4 nM, K_t _OCT2_celltoapical_ = 110.4 nM, V_max_baso_active_ = 3.3 nM/s, K_t_baso_active_ = 29.0 nM, V_max_baso_new_FD_ = 6.0 nM/s, K_t_baso_new_FD_basotocell_ = 628.3 nM* and K_t_baso_new_FD_celltobaso_ = 628.3 nM*. *Kt__baso_new_FD_basotocell_ and Kt__baso_new_FD_celltobaso_ from Figures 8 and 9 did not show a meaningful directional preference, and so the average value of 628.3 nM was used in each direction for the basolateral bidirectional facilitated diffusion transporter.

**FIGURE 11:**
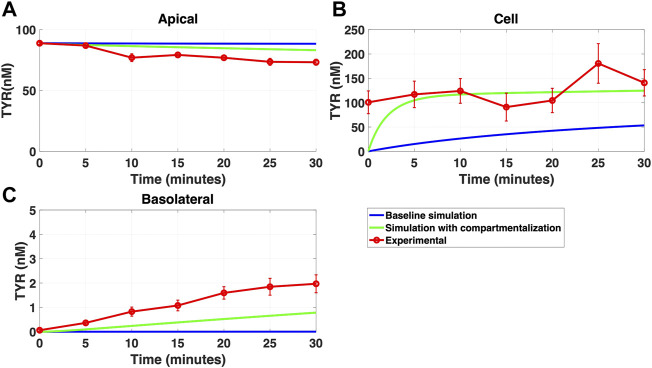
Compartmentalization is needed with introduction of symmetry in the facilitated diffusion transporters with apical loading. The transport of 88.9 nM TYR across Caco-2 cells was modeled following apical loading by solving Eqs 12–14, using the values mentioned in Supplementary Table S2, using MATLAB vR 2021a. Comparisons were made between the models (baseline = blue curve; modified = green curves) and experimental observations; red curve (Sarkar and Berry, 2020) for the apical Panel 11**(A)**, cellular Panel 11**(B)** and basolateral compartments Panel 11**(C)**. When symmetry is introduced in the facilitated diffusion transporters, compartmentalization is needed. Kinetic parameters used for this modelling were: V_max_OCT2_ = 2.3 nM/s, K_t _OCT2_apicaltocell_ = 110.4 nM, K_t _OCT2_celltoapical_ = 110.4 nM, V_max_baso_active_ = 3.3 nM/s, K_t_baso_active_ = 29.0 nM, V_max_baso_new_FD_ = 6.0 nM/s, K_t_baso_new_FD_basotocell_ = 628.3 nM* and K_t_baso_new_FD_celltobaso_ = 628.3 nM*. *Kt__baso_new_FD_basotocell_ and Kt__baso_new_FD_celltobaso_ from Figures 8 and 9 did not show a meaningful directional preference, and so the average value of 628.3 nM was used as the K_t_ in each direction for the basolateral bidirectional facilitated diffusion transporter.

While a compartmentalization factor of 0.36 (64% of intracellular TYR is sequestered into one or more intracellular compartments) allowed a partial matching to experimental observations following basolateral compartment loading (Figures 10A–C), it was not as effective as asymmetric OCT2 transport (c.f. Figure 8), confirmed by generally lower root mean square deviation (RMSD) values for asymmetric OCT2 transport (2.75, 289.76 and 0.9868 for apical, cellular and basolateral compartments respectively) than for compartmentalization model fits (4.23, 228.39 and 1.88, respectively). Similarly following apical loading, while some improvements compared to the baseline model were possible with compartmentalization (RMSD = 7.52, 46.22 and 0.84 for apical, cellular and basolateral compartments; Figures 11A–C), these were not as robust as with asymmetric OCT2 transport (c.f. Figure 9; RMSD = 4.34, 48.86 and 0.26) with RMSD values 2-4 fold lower for asymmetric OCT2 transport in two of the three compartments. We could not attribute significance to the difference in RMSD values for the cellular compartment between the two models, given the large relative uncertainties in concentration in the cellular compartment. However, for both basolateral and apical loading, the OCT2 asymmetry model provided quantitatively better fits for the apical and basolateral concentrations: the RMSD values were approximately twice as small compared to the compartmentalization model. Furthermore, solved kinetics for the required intracellular compartment transporter (V_max_ = 5.2 × 10^–21^ M/s; K_t_ = 154.5 nM; when using Eqs 15–18 were physiologically inconsequential.

## 4 Discussion

The role of TYR in maintaining various physiological functions by activating intracellularly localized TAAR1 has been well established (Berry et al., 2017; Christian and Berry, 2018; Gainetdinov et al., 2018). Previous literature has reported and characterized distinct TYR transport processes that include simple diffusion across synthetic lipid bilayers (Berry et al., 2013), an OCT2-mediated facilitated diffusion transport process across membranes in native neuronal preparations (Berry et al., 2016) and Caco-2 cells (Sarkar and Berry, 2020), and a novel Na^+^-dependent active transporter across the basolateral membrane of Caco-2 cells (Sarkar and Berry, 2020). It is possible other TYR transport processes exist in intestinal cells that were masked due to the active accumulation of TYR across the basolateral Caco-2 cell membrane in this previous study. Hence, here we developed a model based on known transport kinetics, to determine whether these were sufficient to explain previously obtained (Sarkar and Berry, 2020) experimental concentration-time relationships in the Caco-2 model system.

Simulating TYR transport across Caco-2 cells using known transporter kinetics (Figure 1A) did not match experimental concentration-time relationships (Sarkar and Berry, 2020) for basolateral addition (Figures 2A–C). Similarly, for apical addition, this model was unable to recreate experimental concentration-time relationships in each compartment (Figures 3A–C). This suggested the presence of one or more additional TYR transporters in the basolateral membrane. Since OCT2 was already known to be expressed in the apical membrane (Sarkar and Berry, 2020), we first investigated whether adding OCT2 to the basolateral membrane would improve the model. Even if the density of the added basolateral membrane OCT2 was allowed to vary between 0.1x and 20x that in the apical membrane, experimental TYR concentration-time relationships still could not be recreated (Figures 4A–C and 5A–C), suggesting that the additional, basolateral, facilitated diffusion transporter is not OCT2.

We then systematically varied both the V_max_ and K_t_ values for the added basolateral membrane transporter to model a facilitated diffusion transporter with non-OCT2 kinetics. With a V_max_ = 55 nM/s and K_t_ = 203–507.5 nM improvement in modelling accuracy was observed for TYR basolateral addition (Figures 6A–C), but not apical addition (Figures 7A–C). Since model improvements following basolateral loading came at the expense of decreased performance following apical loading, we hypothesized that either asymmetry was needed in one or more of the facilitated diffusion transporters, or that compartmentalization of TYR within the cell was occurring at the high cellular concentrations seen following basolateral loading.

Asymmetry was introduced by allowing K_t_ to vary in each direction of transport for both of the facilitated diffusion transporters, OCT2 in the apical membrane and the added basolateral membrane facilitated diffusion transporter. On introduction of asymmetry, recapitulation of experimental results was possible in all compartments for both apical-to-basolateral and basolateral-to-apical TYR transport (Figures 8A–C and 9A–C). This required an approximate 10-fold preference of OCT2 for transporting TYR into the cell from the apical compartment, while no meaningful directional preference was required for the basolateral membrane facilitated diffusion transporter. The asymmetric transport also allowed for modelling of the marginally higher X_cell_ compared to X_apical_ for apical loading (Figure 9). Interestingly, asymmetric bidirectional facilitated diffusion transporters have previously been described with respect to amino acid transport across the blood brain barrier (Zaragozá, 2020), although we are unaware of any previous reports of directionality exhibited by OCT2. Physiologically this directionality may reflect known differences in pH between the intestinal lumen and cytosolic compartments with typical luminal (apical) pH ranging from 6.0–6.5 while the cytosolic space has a pH of 7.4 (Neuhoff et al., 2005). Such a difference in pH between the apical and cellular compartments, would result in a close to 10-fold difference in the ionization state of TYR between the two compartments. This would be seen as a 10-fold increase in protonated TYR in the luminal space compared to the cytosol suggesting thatOCT2 preferentially binds, and therefore transports, the protonated (positively charged) form of TYR.

As a possible alternative to asymmetry of TYR transport by OCT2, we examined whether the introduction of intracellular compartmentalization of TYR could also recapitulate experimental concentration-time relationships. Previous literature has established an intracellular localization of TAAR1 in various cell types and tissues (Bunzow et al., 2001; Lindemann et al., 2005; Barak et al., 2008; Raab et al., 2016). In particular, endoplasmic reticulum, secretory vesicle (Espinoza et al., 2018), nuclear (Pitts et al., 2019; Barnes et al., 2021) and unspecified intracellular membrane (Miller et al., 2005) localizations have been suggested. Accumulation of TYR in one or more of these compartments could therefore be a requirement for TAAR1 binding to occur. Initially we simply introduced a compartmentalization factor, ‘z’, where ‘z’ represents the fraction of intracellular TYR available to cross basolateral and apical membranes. A value of z = 0.36, indicating 64% of TYR was sequestered into one or more subcellular compartment(s), was obtained which partially alleviated the need for asymmetry of OCT2 transport, at least following basolateral loading. With 64% of TYR sequestered into a compartment comprising 10% of the cellular volume, this would suggest the presence of an active transporter in the intracellular compartment. Although modelling closely matched experimental for some compartments (Figures 10, 11) the match to experimental was visibly less close than that obtained with asymmetric transport (Figures 8, 9) and had 2–4 fold higher RMSD values in two of the three compartments. Further, the compartmentalization appeared to provide a better fit for basolateral, compared to apical loading, suggesting that a compartment transporter would primarily only be active at the higher intracellular concentrations seen following basolateral loading. We next sought to determine the kinetic parameters that would be required for transport into such an intracellular compartment(s) under the assumption that the volume of the inner compartment was 10% (or less) of the total volume of the cell. Although a physiologically relevant K_t_ value (154.5 nM) was predicted which would be consistent with activity primarily at the higher concentrations seen following basolateral transport, the V_max_ (5.2 × 10^–21^ nM/s) is equivalent to a mere 86 molecules per second per cell transported, indicating an unrealistically low density of transporter to be of physiological relevance.

With our previous observation that there is active accumulation of TYR within the cells following basolateral addition (Sarkar and Berry, 2020), and the observed high K_t_ of the basolateral membrane facilitated diffusion transporter (Table 1), we propose that this is not a selective TYR transporter but rather non-selectively includes TYR in its substrate spectrum at the high intracellular concentrations seen due to the active transporter. While the current modelling suggests the need for additional low affinity transporters to be present, it provides limited details on the potential identity of such transporters. TYR is a substrate for both Organic Cation Transporter 3 (OCT3; Slc22A3) and Plasma Membrane Monoamine Transporter (PMAT; Slc29A4) with a reported K_t_ at each of approximately 280 nM (Engel and Wang, 2005; Chen et al., 2010), not too dissimilar from our modelled K_t_ of approximately 600 nM. Further, both have been reported to be expressed in the small intestine (Shirasaka et al., 2016; Dawed et al., 2019), making these potential candidates for the additional basolateral TYR facilitated diffusion transporter required in our modelling. The high variability of measured intracellular TYR concentrations as a result of the very small volume of the cellular compartment, introduces considerable uncertainty in the estimation of rates of change of intracellular TYR and consequently deduced kinetics. Identification of candidate transporters will therefore require further studies to identify the TYR binding proteins present in Caco-2 cell preparations. This transporter could become important pathologically during compromised TYR metabolism, such as following treatment with MAO inhibitors where it would provide a conduit for dietary TYR to enter the bloodstream when present at high levels. Such excess levels of TYR are known to cause indirect sympathomimetic effects leading to severe hypertensive crisis and in extreme situations, death (Finberg and Gillman, 2011).

The studies reported here are likely to be generally relevant to other cell types beyond Caco-2 cells. Broad substrate facilitated diffusion transporters such as OCT3 (Wu et al., 2000; Lips et al., 2005) and PMAT (Wang, 2016) are known to be present in the lung epithelia, brain, hepatocytes, kidney, and heart, and at least some of these are also known to express TAAR1 (Borowsky et al., 2001; Lindemann et al., 2005; Revel et al., 2012; Cisneros and Ghorpade, 2014; Fleischer et al., 2018). In particular, cells associated with barrier functions such as the blood-brain-barrier and fetal-placental barrier express a broad range of broad-spectrum transporters. Interestingly, TAAR1 has been implicated in recurrent miscarriages (Stavrou et al., 2018), and as such identifying the transporters that regulate endogenous agonist passage across the placental barrier may be an avenue to therapeutic options in susceptible individuals.

## 5 Conclusion

This study provides the first evidence for an additional basolateral membrane facilitated diffusion transporter present in Caco-2 cells that includes TYR in its substrate spectrum. This additional transporter does not have kinetic characteristics consistent with those of OCT2, and may represent either OCT3 or PMAT. Rather than selectively targeting TYR for transport we propose that TYR is transported non-selectively when present at high concentrations (>250 nM). In addition, our modelling predicts that the apical membrane OCT2 shows an unequal bidirectional transport of TYR which we hypothesize is reflective of the pH differences between the intestinal lumen and epithelial cell cytosol.

## Data Availability

The original contributions presented in the study are included in the article/Supplementary Material, further inquiries can be directed to the corresponding author.
