# Supplementary material for: Modelling of p-tyramine transport across human intestinal epithelial cells predicts the presence of additional transporters
Source: Front Physiol. 2022 Nov 10;13:1009320. doi: 10.3389/fphys.2022.1009320 (PMC9733674; doi:10.3389/fphys.2022.1009320)
Supplement: Supplementary file 1 [file Table1.docx]

| **Parameter** | **Experimentally determined kinetics** | **Parameters used for figures 2, 3** | **Parameters used for figures 4, 5** | **Parameters used for figures 6 7** | **Parameters used for figures 8, 9** | **Parameters used for figures 10, 11** |
| --- | --- | --- | --- | --- | --- | --- |
| V_max_OCT2_ | 0.1 nM/s | 0.1 nM/s | 0.1 nM/s | 0.1 nM/s | 2.3 nM/s | 2.3 nM/s |
| K_t _OCT2_apicaltocell_ | 101.5 nM | 101.5 nM | 101.5 nM | 101.5 nM | 110.4 nM | 110.4 nM |
| K_t _OCT2_celltoapical_ |  | n/a | n/a | n/a | 1227.9 nM | 110.4 nM |
| V_max_baso_active_ | 43.0 nM/s | 43.0 nM/s | 43.0 nM/s | 43.0 nM/s | 3.3 nM/s | 3.3 nM/s |
| K_t_baso_active_ | 33.1 nM | 33.1 nM | 33.1 nM | 33.1 nM | 29.0 nM | 29.0 nM |
| V_max_baso_new_FD_ | n/a | n/a | 0.01-2 nM/s | 50 nM/s | 6.0 nM/s | 6.0 nM/s |
| K_t_baso_new_FD_basotocell_ | n/a | n/a | 101.5 nM | 101.5-1015 nM | 584.1 nM^*^ | 628.3nM^*^ |
| K_t_baso_new_FD_celltobaso_ |  |  | n/a | n/a | 672.4nM^*^ | 628.3 nM^*^ |

**Supplementary material**

**Supplemental Table 1: Kinetic parameters used for each model scenario.** Kinetic parameter used for OCT2 and the added basolateral membrane transporter for different model scenarios of TYR transport across Caco-2 cells.*K_t_baso_new_FD_basotocell_ and K_t_baso_new_FD_celltobaso_ did not show a meaningful directional preference, and so for figures 12 and 13 the average value of 628.3 nM was used as the K_t_ in each direction for the basolateral bidirectional facilitated diffusion transporter.
